# Growth of Continuous Monolayer Graphene with Millimeter-sized Domains Using Industrially Safe Conditions

**DOI:** 10.1038/srep21152

**Published:** 2016-02-17

**Authors:** Xingyi Wu, Guofang Zhong, Lorenzo D'Arsié, Hisashi Sugime, Santiago Esconjauregui, Alex W. Robertson, John Robertson

**Affiliations:** 1 Department of Engineering, University of Cambridge, Cambridge, CB 3 0FA, United Kingdom; 2 Department of Materials, University of Oxford, Oxford, OX 1 3 PH, United Kingdom

## Abstract

We demonstrate the growth of continuous monolayer graphene films with millimeter-sized domains on Cu foils under intrinsically safe, atmospheric pressure growth conditions, suitable for application in roll-to-roll reactors. Previous attempts to grow large domains in graphene have been limited to isolated graphene single crystals rather than as part of an industrially useable continuous film. With both appropriate pre-treatment of the Cu and optimization of the CH_4_ supply, we show that it is possible to grow continuous films of monolayer graphene with millimeter scale domains within 80 min by chemical vapour deposition. The films are grown under industrially safe conditions, i.e., the flammable gases (H_2_ and CH_4_) are diluted to well below their lower explosive limit. The high quality, spatial uniformity, and low density of domain boundaries are demonstrated by charge carrier mobility measurements, scanning electron microscope, electron diffraction study, and Raman mapping. The hole mobility reaches as high as ~5,700 cm^2^ V^−1^ s^−1^ in ambient conditions. The growth process of such high-quality graphene with a low H_2_ concentration and short growth times widens the possibility of industrial mass production.

Due to its outstanding properties^1^, graphene has many promising applications in electronics^2^, photonics^3^, sensors^4^, energy generation and storage^5^. Cu-catalyzed chemical vapor deposition (Cu-CVD) of graphene emerged as the most promising synthesis method due to its scalability, accurate control of monolayer uniformity, potential for uniform coverage, and compatibility with integrated manufacturing^6^,^7^,^8^. To date, the growth of continuous graphene films on Cu has been achieved under both low and atmospheric pressures^6^,^9^,^10^, up to 100 meter-scale using a roll-to-roll apparatus^11^, at reduced temperature (<420 °C)^12^, and a rapid growth rate^13^. However, a common problem with the current continuous graphene films lies in their polycrystalline nature with typical domain sizes limited below ~300 μm^13^,^14^,^15^,^16^. The domain boundaries associated with polycrystalline graphene are undesirable as they degrade graphene electronic quality, mechanical strength, thermal conductivity, and oxidation resistance^17^,^18^,^19^,^20^,^21^. To reduce the number density of graphene domain boundaries, the domain sizes have to be further increased. Recently, millimeter to centimeter-sized graphene single crystals have been successfully produced by modified Cu-CVD methods, which include Cu electropolishing^15^,^22^,^23^,^24^, ~7 h reductive annealing of Cu^23^, high temperature re-solidifying of Cu^25^, surface oxygen-assisted growth^26^,^27^,^28^,^29^,^30^, and Cu enclosure structures^31^. However, the graphene synthesized by these methods only has limited coverage with randomly positioned domains making them unsuitable for large scale device manufacture, which requires the high yield of continuous graphene. Another problem with these methods is that they normally have unacceptably long growth times (5–48 h).

In addition, the safety issue of the CVD process is usually ignored, despite its importance in industrial production. Further economic development would desire the growth at large scale by roll-to-roll reactors, preferentially under intrinsically safe and atmospheric pressure conditions. The flammability of H_2_ at typical growth conditions is a particular problem (the explosive limit of H_2_ is 4–75% (volume concentration) at room temperature in air). Although there are several reports on graphene growth using no H_2_^13^,^32^,^33^ or diluted H_2_^22^,^29^,^34^,^35^,^36^,^37^,^38^,^39^, their charge carrier mobilities are limited below ~4, 000 cm^2^V^−1^s^−1^, which is not qualified for “electronic-grade” applications^8^. Besides, H_2_-free growth removes a useful second parameter for control over graphene quality, coverage, and domain size. Thus, the growth of graphene with a high charge carrier mobility is still a challenge under industrially safe CVD conditions.

In this paper, we demonstrate the growth of continuous monolayer graphene with millimeter-sized domains using diluted H_2_ (2%) in 80 min. The average domain size reaches ~1 mm, which is the largest achieved to date for continuous graphene. The hole mobility reaches as high as 5,700 cm^2^V^−1^s^−1^, the highest value among the reported mobilities using similarly low concentrations of H_2_. We achieve this by combining electropolishing and non-reductive annealing of the Cu foils with optimizing the CH_4_ concentration. This work brings the current growth method of graphene a large step towards scalable and safe production.

## Results

### Growth of continuous monolayer graphene films

**Figure 1:**
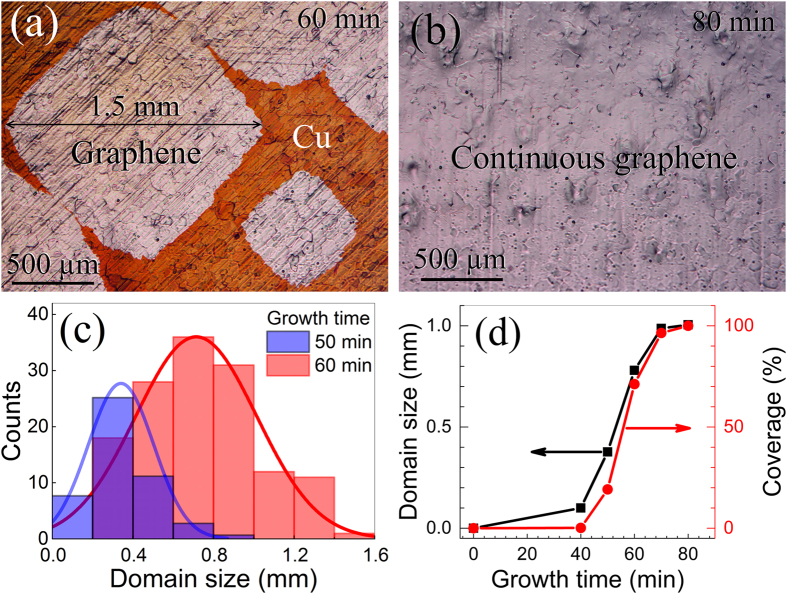
Characterization of graphene coverage and domain size. (**a,b**) Optical microscope images of graphene grown on Cu foils after 60 min and 80 min, respectively. Growth conditions: 1030 °C, 0.30 sccm CH_4_, 80 sccm H_2_, 3,920 sccm Ar. (**c**) Histograms of domain size for 50 min and 60 min growth. (**d**) The graphene coverage and the average domain size as a function of growth time.

We use electropolished Cu foils as the catalyst for the graphene growth. The Cu foil is annealed under pure Ar after the temperature ramps to 1030 °C under pure Ar. The graphene growth is carried out by simultaneously introducing CH_4_ and H_2_ that are diluted to well below their low explosive limit (LEL). Fig. 1a,b show the optical microscope (OM) images of graphene grown on Cu for 60 and 80 min under 75 ppm (parts per million) CH_4_, respectively. The as-grown samples are baked in air (at 200 °C for 1 min) to visualize the graphene-covered regions. In 60 min, the graphene partly covers the Cu substrate with ~70% coverage. Some isolated domains reach as large as ~1.5 mm (Fig. 1a). After 80 min, the baked sample shows no colour change and no colour contrast (Fig. 1b) indicating that the Cu surface is fully covered and protected by graphene from oxidation. The histograms in Fig. 1c show detailed distributions of domain sizes of graphene grown in 50 and 60 min. Both the average and distribution of the domain sizes increase with the growth time; the average sizes are 0.4 and 0.8 mm for 50 and 60 min, respectively. The time evolution of the average domain size and coverage are plotted in Fig. 1d. The estimated average domain size at full coverage (80 min) reaches 1.0 mm. The coverage follows a typical sigmoidal curve, featuring an incubation period during 0–40 min, a linear growth period during 50–60 min, and a slowing down period during 70–80 min^40^.

**Figure 2:**
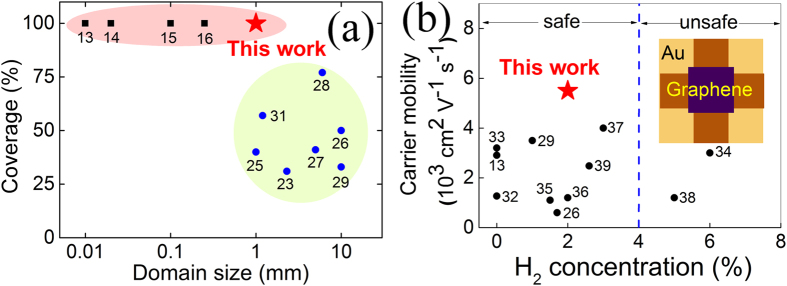
A summary of (a) graphene coverage versus domain size and (b) charge carrier mobility versus the highest H_2_ concentration used during synthesis processes. (**a**) Squares: full coverage with limited domain size (≤300 μm). Circles: large single crystals (1–10 mm) with limited coverage. Star: this work. (**b**) Circles: literature work. Star: this work. Dashed line marks the H_2_ lower explosive limit (~4%). Inset: Schematic for Hall device of Van der Pauw geometry for mobility measurement in this work. The digital number adjacent to each data point gives the reference number.

Compared to the published results on continuous graphene^13^,^14^,^15^,^16^ (squares in Fig. 2a), our average domain size (~1 mm) is at least three times larger. As the density of domain boundaries is inversely proportional to the domain area, this means the domain boundary density of our graphene films is at least an order of magnitude lower than previously shown. Although the domain size in our work is not as large as that of centimeter-scale graphene single crystals (circles in Fig. 2a), the full coverage of our graphene makes it more suitable for large scale graphene device manufacture. In addition, the concentration of H_2_ throughout the whole process is controlled to be no more than 2%, which is much lower than that used in the growth of large single crystals^26^,^28^.

### Characterization

The quality of the graphene is assessed by charge carrier mobility measurements, scanning electron microscope (SEM), transmission electron microscope (TEM), OM, and Raman spectroscopy.

The charge carrier mobility of the as-grown continuous graphene film is measured by Hall effect in the form of a Van der Pauw structure^41^ (the inset in Fig. 2b). The hole mobility in ambient conditions reaches 5,500 ± 200 cm^2^V^−1^s^−1^ (mean ± standard deviation) based on the Drude model^41^ and the carrier concentration is about 1.6 × 10^12^ cm^−2^. As a reference, we also measure a graphene sample grown by the typical low pressure CVD using H_2_ annealing as well as a commercial graphene sample. The mobility and carrier concentration of the reference samples are (4,100 cm^2^V^−1^s^−1^, 1.6 × 10^12^ cm^−2^) and (3,400 cm^2^V^−1^s^−1^, 9.0 × 10^11^ cm^−2^) respectively. Compared to the reference samples and the reported values for continuous monolayer graphene, the mobility of our graphene is notably higher than all other results using similarly low concentrations of H_2_ (0–6%, see Fig. 2b)^13^,^22^,^29^,^32^,^33^,^34^,^35^,^36^,^37^,^38^,^39^. The high mobility combined with the intrinsically safe CVD conditions makes our method particularly suitable for the large scale production of “electronic-grade” graphene films. In addition, the sample size in our mobility measurement (50 × 50 μm^2^) is significantly larger than the typical device sizes reported to date(~10 × 10 μm^2^)^41^; a high mobility over such a large area demonstrates the high quality and spatial uniformity of the graphene.

**Figure 3:**
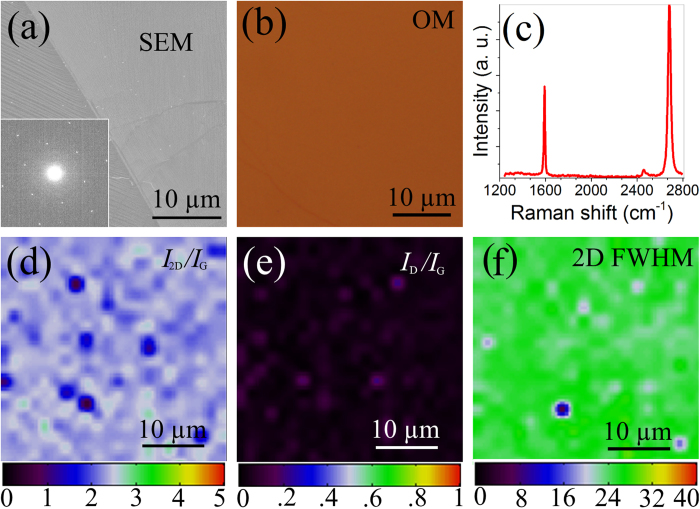
Characterization of graphene quality. (**a**) Scanning electron microscope image of graphene grown on Cu foil. Inset: electron diffraction pattern by transmission electron microscope. (**b**) Optical microscope image of graphene transferred onto SiO_2_ (300 nm) /Si. (**c**) Typical Raman spectrum of transferred graphene. Raman maps of (**d**) 2D to G peak intensity ratio, (**e**) D to G peak intensity ratio and (**f**) 2D FWHM over a 40 × 40 μm^2^ area. The unit for the colour bar in (**f**) is cm^−1^.

The SEM image in Fig. 3a shows the macroscopic uniformity of a monolayer graphene film grown on Cu. The OM image of the graphene transferred onto SiO_2_ in Fig.3b confirms the uniformity is preserved after the transfer. Figure 3c shows a typical Raman spectrum measured from the transferred graphene. It shows an intense 2D peak at 2,682 cm^−1^, a G peak at 1,592 cm^−1^ and no detectable D peak (~1,350 cm^−1^). The intensity ratio of the 2D peak to the G peak (*I*_2D_*/I*_G_) is ~2. The 2D peak is of Lorentzian shape with one single component and the full width at half maximum (FWHM) is ~26 cm^−1^. These are the fingerprints of high quality monolayer graphene^42^. The inset in Fig. 3a shows a typical electron diffraction pattern of such a graphene film, displaying a six-fold symmetry that is characteristic of graphene. The intensity ratio of the outer diffraction spot over the inner spot is close to 0.5, also confirming the monolayer nature.

To further confirm the homogeneity of the continuous graphene film, we carry out Raman mapping^17^ over a 40 × 40 μm^2^ area. The *I*_2D_*/I*_G_, *I*_D_*/I*_G_, and FWHM of the 2D peak reaches 2.3  ±  0.2 (mean ± standard deviation, Fig. 3d), 0.04 ± 0.02 (Fig. 3e), and 25 ± 2 cm^−1^ (Fig. 3f), respectively. The mapping results indicate the spatial uniformity, high quality, and monolayer nature of the graphene.

## Discussion

We now discuss the essential factors for synthesizing the continuous graphene films while still achieving millimeter-sized domains under 2% H_2_ in 80 min. Firstly, we electropolish Cu foils to reduce the surface roughness. Supplementary Fig. S1 compares the surface roughness of a Cu foil before and after electropolishing. The top panel gives the mapping images obtained by an optical profilometer (OP) and the bottom panel shows the corresponding line profiles marked in the top panel. As seen in Supplementary Fig. S1a, the as-received Cu foil has high density of rolling-induced sharp protrusions and grooves. Electropolishing can efficiently remove these features and obtain a much smoother surface (Supplementary Fig. S1b). The root mean square (RMS) roughness (*R*_rms_) drops from 320–100 nm over a 50 μm range as shown in Supplementary Fig. S1c,d.

**Figure 4:**
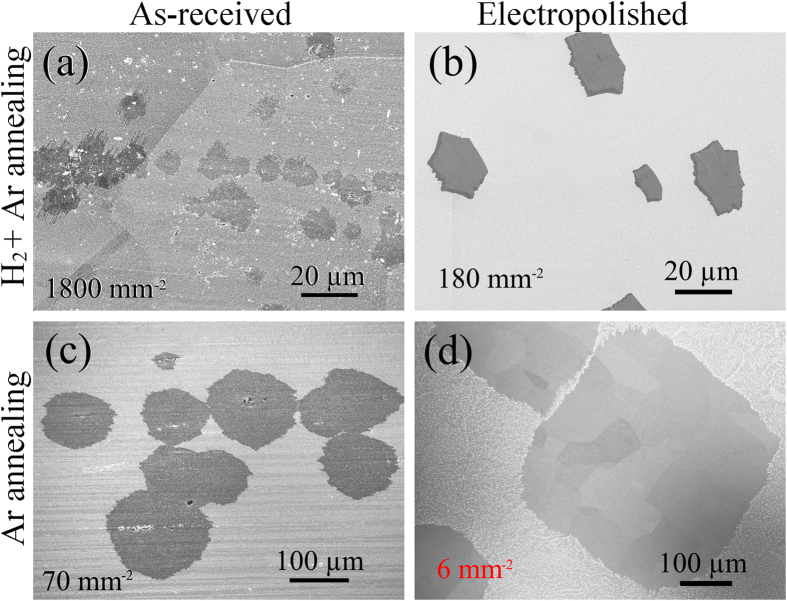
Effects of electropolishing and non-reductive annealing on graphene nucleation. Scanning electron microscope image of graphene grown on Cu which have been (**a**) annealed by H_2_ + Ar, (**b**) electropolished and annealed by H_2_ + Ar, (**c**) annealed by Ar, and (**d**) electropolished and annealed by Ar. Growth conditions: 1030 °C, 2% H_2_, 100 ppm CH_4_, 20 min for (**a**) and (**b**); 1030 °C, 2% H_2_, 100 ppm CH_4_, 40 min for (**c**) and (**d**). Nucleation densities are ~1,800 mm^−2^, ~180 mm^−2^, ~70 mm^−2^, and ~6 mm^−2^, respectively.

The effect of electropolishing on graphene nucleation is shown in Fig. 4. The graphene preferentially nucleate along rolling grooves on the as-received Cu foils as seen in Fig. 4a,c, regardless of the annealing atmosphere. In contrast, the nucleation is much more uniform on the polished Cu foils, as seen in Fig. 4b,d. The nucleation density drops by a factor of ~10, comparing Fig. 4a with 4b, 4c, with 4d. As crystal defects on the surface rough regions provide active sites for graphene nucleation^43^, electropolishing can lower the nucleation density by removing these defects and thus promote a uniform nucleation. Although some pits caused by electropolishing can be seen in Fig. 1b, we do not observe any significant effect of these pits on the nucleation of graphene. Supplementary Fig.S2 shows the effect of electropolishing time on the nucleation of graphene. As can be seen, a polishing time longer than 20 min is necessary under our conditions to efficiently remove the manufacturing-induced rolling grooves, which seem to be the most significant factor affecting the nucleation uniformity and density of graphene.

Secondly, we anneal the Cu foils in non-reductive atmosphere (pure Ar) which is found to drastically reduce the nucleation density, by around 30-fold, compared with annealing in reductive gas (H_2_ diluted in Ar), as can be seen in Fig. 4. The non-reductive annealing yields single crystals as large as 100 μm even on non-polished Cu (Fig. 4c). As the catalytic ability of oxidized Cu is weaker than that of metallic Cu^26^, non-reductive annealing that keeps Cu oxide on the surface can suppress graphene nucleation. A combinational use of the electropolishing and the non-reductive annealing reduces the nucleation density to the order of several nuclei per mm^2^ (Fig. 4d). Thus the domain size grown on electropolished Cu using non-reductive annealing is about 20 times larger than that on non-polished Cu using reductive annealing.

Finally, we optimize the CVD parameters towards obtaining even larger domains. In order to determine a proper H_2_ concentration, we have investigated the effect of H_2_ concentration on graphene nucleation density, as shown in Supplementary Fig. S4. It can be seen that the graphene nucleation density increase slightly with the reduction of H_2_ from 4% to 2%. However, the density increases about 2 orders of magnitude when decreasing H_2_ from 2% to 1%. We finally choose a H_2_ concentration of 2% as our growth condition. This is to dramatically reduce the nucleation density of graphene but allow the subsequent growth of continuous graphene. As the LEL of H_2_ is 4%, the use of 2% H_2_ can also avoid some marginal safety uncertainties. Once the H_2_ concentration is fixed at 2%, we find that decreasing the CH_4_ concentration can also tune the nucleation density of graphene. Supplementary Fig. S4 shows the dependence of the nucleation density on the CH_4_ concentration over the range of 75–200 ppm. The nucleation density is measured at ~70% coverage for each condition. The dependence is roughly linear, indicating the CH_4_ concentration is also an efficient and stable parameter to tune the nucleation density. We then choose 75 ppm as an optimal CH_4_ concentration which yields continuous graphene film with millimeter-sized domains in 80 min growth time. Although even lower CH_4_ concentrations can potentially achieve larger domains, it takes much longer time to reach full coverage, which is less preferable in the mass production.

## Conclusion

In summary, we have grown high quality continuous monolayer graphene films on Cu foils with millimeter-sized domains and a charge carrier mobility as high as 5,700 cm^2^V^−1^s^−1^ using a safe concentration of H_2_ in 80 min. This is the first time that the average domain size of continuous graphene films reaches millimeter scale. The domain size grown on electropolished Cu using non-reductive annealing is about 20 times larger than that on non-polished Cu using reductive annealing. This result helps improve the scalability and the safety of industrial production of high quality graphene.

## Methods

### Cu electropolishing

Commercial Cu foils (Alfa Aesar, 99.8% purity, 25 μm thickness) are electropolished in a home-built cell. Two pieces of 10  ×  10 cm^2^ Cu foils separated by 5 cm are used as an anode and a cathode, and 85% H_3_PO_4_ solution is used as the electrolyte. The anodic Cu foil is electropolished at 1.9 V for 30 min in ambient conditions and then rinsed by isopropyl alcohol and de-ionized water.

### Graphene synthesis

The polished Cu foil is loaded into a 2-inch quartz tube furnace, which is then heated up to 1030 °C at ~20 °C/min under 1 atm with 400 sccm (standard cubic centimeter per minute) Ar. The Ar flow rate is then increased to 4,000 sccm to anneal the Cu for 5 min. Graphene growth is carried out by flowing 3,920 sccm Ar, 80 sccm H_2_ (2%), and 0.20–0.80 sccm CH_4_ (50–200 ppm) for 10–80 min, followed by cooling down to room temperature under the same Ar and H_2_ flow (without CH_4_). The gas pressure is maintained at 1 atm.

### Statistics of nucleation density and domain size

The post-growth graphene on Cu foils are baked at 200 °C for 1 min in air on a hot plate to oxidize the Cu areas that are not covered by graphene. The colour contrast between the graphene-covered area and exposed area can visualize individual domains^44^. The nucleation density and domain size are derived from OM images of the baked samples. For low coverage graphene (≤70%), the average domain size is calculated as the RMS of the diagonal lengths of all the domains counted from OM images. For high coverage graphene (70−100%), the average domain size is estimated using the nucleation density counted at ~70% coverage as the nucleation rate has declined to almost zero over this coverage.

### Graphene transfer and characterization

For charge carrier mobility measurement, the graphene film is transferred onto a SiO_2_(300 nm)/Si wafer using a poly(methyl methacrylate) support layer and 0.05 M aqueous solution of (NH_4_)_2_S_2_O_8_ as Cu etchant^45^. Then Hall devices with Van der Pauw geometry are fabricated on SiO_2_ (300 nm) /Si wafers with 50 × 50 μm^2^ graphene area patterned by electron-beam lithography. Metal electrodes (45 nm Au /5 nm Ni) are deposited by thermal evaporation. The carrier mobility is measured from six devices under 1T magnetic field at room temperature using Hall and Van der Pauw Measurement System of MMR Technologies.

The samples are characterized by optical microscope (Nikon ECLIPSE LV150N), scanning electron microscope (Philips XL30, 1 kV), transmission electron microscope (JEOL 2100, 80 kV), Raman spectroscopy (Renishaw InVia spectrometer, 514 nm excitation), and optical profilometer (Wyko NT1100). For Raman characterization, the graphene film is transferred onto a SiO_2_(300 nm)/Si wafer. For electron diffraction study, the graphene film is transferred onto a 3 mm-diameter holey carbon copper grid (Agar Scientific No. AGS147).

## Additional Information

**How to cite this article**: Wu, X. *et al.* Growth of Continuous Monolayer Graphene with Millimeter-sized Domains Using Industrially Safe Conditions. *Sci. Rep.* **6**, 21152; doi: 10.1038/srep21152 (2016).

## Supplementary Material

Supplementary Information
