# Supplementary material for: Growth of Continuous Monolayer Graphene with Millimeter-sized Domains Using Industrially Safe Conditions
Source: Sci Rep. 2016 Feb 17;6:21152. doi: 10.1038/srep21152 (PMC4756286; doi:10.1038/srep21152)
Supplement: Supplementary Information [file srep21152-s1.pdf]

Supplementary Information

Growth of Continuous Monolayer Graphene with Millimeter-sized Domains Using Industrially Safe Conditions

Xingyi Wu[1], Guofang Zhong[1,*], Lorenzo D'Arsié[1], Hisashi Sugime[1], Santiago Esconjauregui[1], Alex W. Robertson[2], and John Robertson[1]

[1]*Department of Engineering, University of Cambridge, Cambridge, CB3 0FA, United Kingdom*

[2]*Department of Materials, University of Oxford, Oxford, OX1 3PH, United Kingdom*

*To whom correspondence should be addressed. Email: gz222@cam.ac.uk

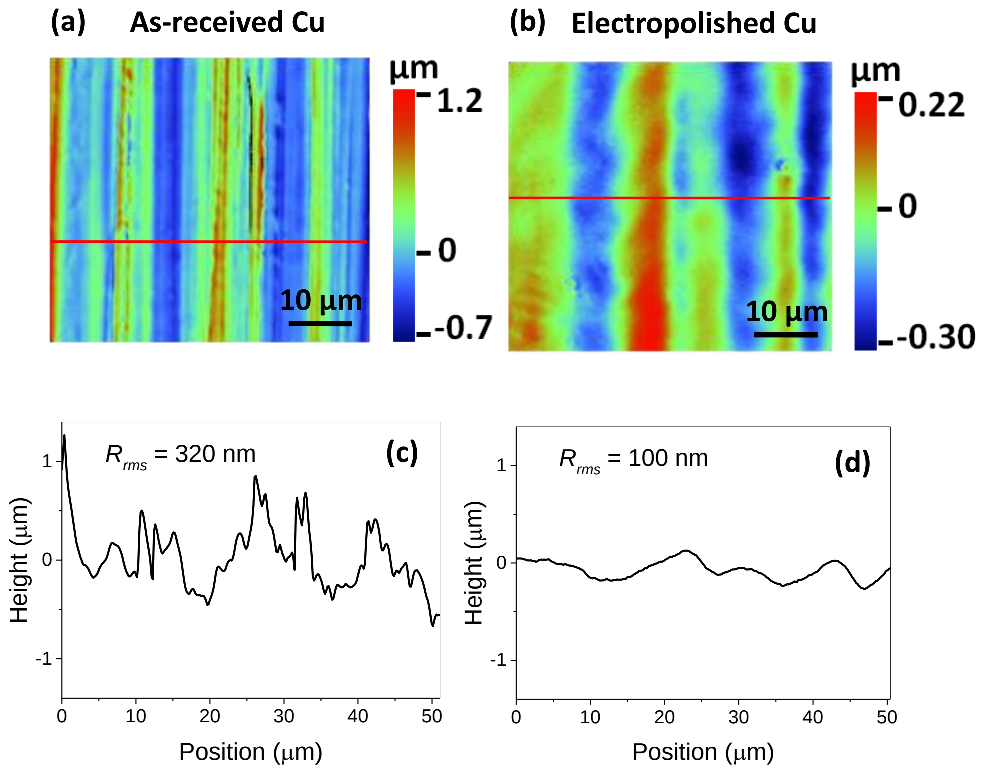

Supplementary Figure S1. Cu surface morphology before and after electropolishing. Optical profilometry mapping of (a) as-received and (b) electropolished Cu foil surfaces. Line profile of surface roughness of (c) as-received and (d) electropolished Cu foil, corresponding to the red lines marked in (a) and (b), respectively.

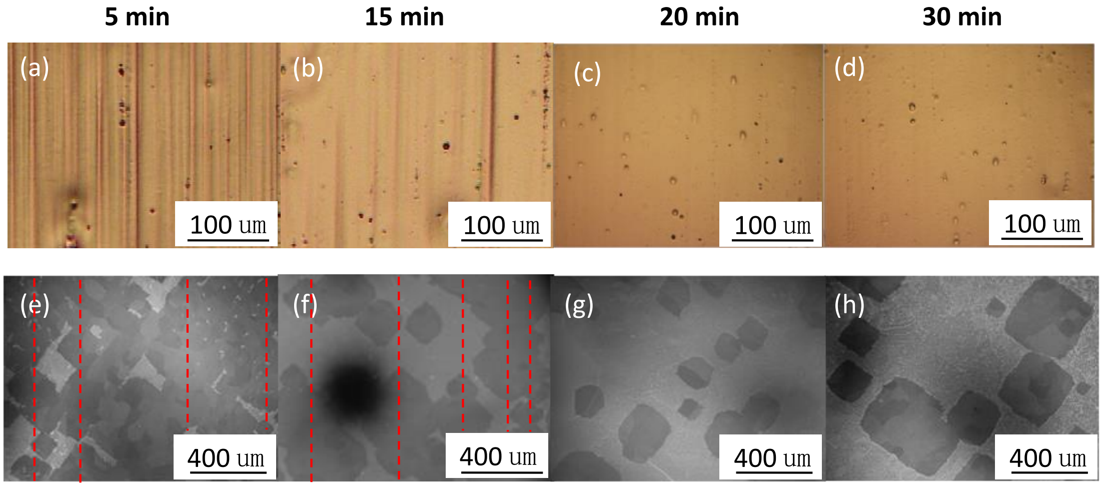

Supplementary Figure S2. Effect of electropolishing time on graphene nucleation. (a – d) OM images of Cu foils electropolished in 5-30 min; (e – h) SEM images of the as-grown graphene on Cu foils corresponding to (a – d), respectively. Red dashed lines indicate the orientations of the rolling grooves. Growth conditions: 1030ºC, 100 ppm CH_4, 40 min.

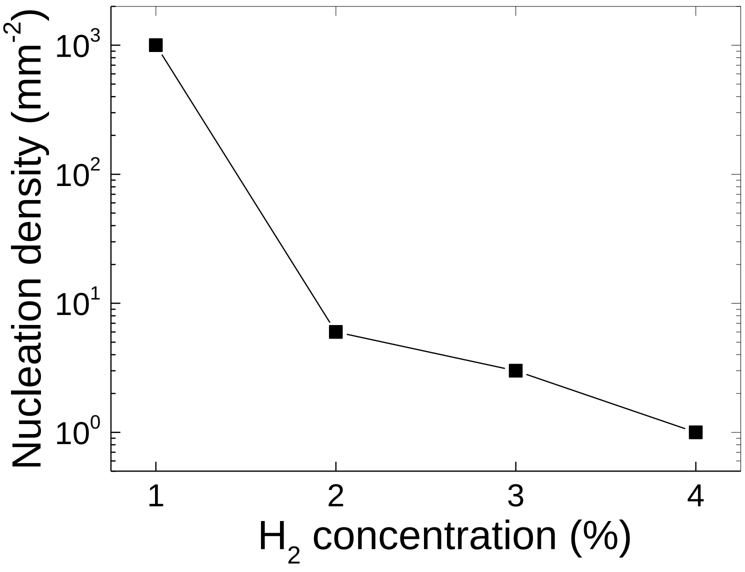

Supplementary Figure S3. Dependence of graphene nucleation density on H2 concentration. Growth conditions: 1030°C, 100 ppm CH_4.

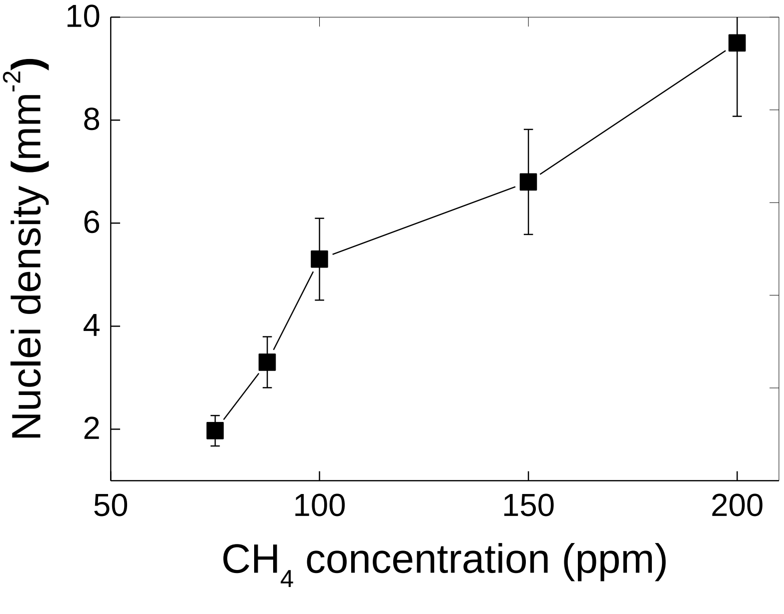

Supplementary Figure S4. Dependence of nucleation density on CH_4 concentration using electropolished Cu foil and Ar annealing.
